# What Has Replication Ever Done for Us? Insights from Neuroimaging of Speech Perception

**DOI:** 10.3389/fnhum.2017.00041

**Published:** 2017-02-01

**Authors:** Samuel Evans

**Affiliations:** ^1^Institute of Cognitive Neuroscience, University College LondonLondon UK; ^2^Department of Psychology, University of WestminsterLondon, UK

**Keywords:** replication, speech perception, intelligibility, superior temporal sulcus, MVPA, fMRI, PET, neuroimaging

Replication of a previous scientific finding is necessary to verify its truth. Despite the importance of replication, incentive systems in science favor novel findings over reliable ones. Consequently, little effort is devoted to reproducing previous results compared to finding new discoveries. This is particularly true of brain imaging, in which the complexity of study design and analysis, and high costs and time intensive data collection, act as additional disincentives. Unfortunately, functional imaging studies often have small sample sizes (e.g., *n* < 20) resulting in low statistical power and inflated effect sizes, making them less likely to be successfully reproduced (Carp, 2012; Button et al., 2013; Szucs and Ioannidis, 2016; Poldrack et al., 2017). This, in addition to discovered errors in analysis software (Eklund et al., 2016; Eickhoff et al., 2017) and wider concerns about the reliability of psychological research (Simmons et al., 2011; Open Science Collaboration, 2015), has led to a crisis of confidence in neuroscientific findings. Recent work has begun to address issues around the reproducibility of brain imaging (see Barch and Yarkoni (2013) for an introduction to a special issue). Indeed, there have been some notable successes, for example, in identifying features of study design and analysis that influence reproducibility (Bennett and Miller, 2013; Turner and Miller, 2013), as well as in the development of tools to facilitate data sharing (Poldrack et al., 2013; Gorgolewski et al., 2016b), to evaluate data reliability (Shou et al., 2013) and to aid the reporting and reliability of data processing and analysis (Poldrack et al., 2008; Carp, 2013; Pernet and Poline, 2015; Gorgolewski et al., 2016a). However, despite these advances, relatively few functional imaging replication studies have been conducted to date. Recently in the speech perception domain, there have been some notable replication attempts, here I discuss what has been learnt from them about speech perception and the replication endeavor more generally.

Defining replication is difficult as replications can take different forms. A broad distinction exists between direct replication, in which an identical procedure is repeated with the aim to recreate the previous experiment in its entirety, and conceptual replication, in which a previous result or hypothesis is tested with different methods (Schmidt, 2009). There have been a number of recent *conceptual* replication attempts in the field of speech perception research. As might be expected, the outcome of these studies has been mixed. For example, Arsenault and Buchsbaum (2016) failed to replicate evidence for somatotopic mapping of place of articulation distinctions in response to hearing spoken syllables, a finding originally demonstrated by Pulvermüller et al. (2006). This finding was controversial, with the original authors suggesting that differences in methodology explained the failure to replicate (Schomers and Pulvermüller, 2016). Whilst failures to replicate have become newsworthy, successful replications are sometimes perceived as less noteworthy, despite the fact that they often provide new knowledge, as well as confirming what was already known. Here, I describe in detail the outcome of successful replications of a paradigm investigating the neural basis of spoken sentence comprehension (Scott et al., 2000). This paradigm has been replicated several times, twice by researchers associated with the original study (Narain et al., 2003; Evans et al., 2014) and once by an independent group (Okada et al., 2010) (see Table 1 for a summary of the studies). Using these studies as an example, I demonstrate how advances in methodology in combination with replication have advanced our understanding of the neural systems supporting speech perception.

**Table 1:** **A summary of the features of the different studies**.

**Study**	**Imaging modality**	**Participants**	**Inference**	**Analysis**	**fMRI scanning protocol**	**Software**	**Multiple comparison correction**	**Task**	**Stimuli**
Scott et al., 2000	PET	8	Fixed effects	Univariate		SPM 99	Peak level Family Wise Error (FWE) corrected, *p* < 0.05	Passive	Southern British English (SBE)—Bamford Kowal Bench (BKB) sentences (Bench et al., 1979)^a^
Narain et al., 2003	fMRI	11	Fixed effects	Univariate	Sparse sampling	SPM 99	Peak level FWE corrected, *p* < 0.05	Passive	SBE BKB sentences
Okada et al., 2010	fMRI	20	Random effects	Univariate & MVPA	Continuous acquisition	AFNI	Peak level False Discovery Rate (FDR) corrected, *q* < 0.05	Active Button press (Intelligible? Y/N)	American English BKB sentences
Evans et al., 2014	fMRI	12	Random effects	Univariate & MVPA	Sparse sampling	SPM8	Peak level *p* < 0.001 uncorrected, FDR cluster corrected, *p* < 0.05	Passive	SBE BKB sentences

a *It is not possible to know exactly which individual BKB sentences were used in each study as this was not reported. The exact BKB sentences used are likely to differ across studies*.

The original Scott et al. study is influential. To date it has received 921 Google scholar citations (Scholar.google.com., 2017) and has played an important role in shaping models of speech processing (Scott and Johnsrude, 2003; Scott and Wise, 2004; Rauschecker and Scott, 2009). Prior to this, researchers typically compared neural activity elicited by speech to activity evoked by simple sounds like tones or noise bursts. These sounds underestimated the complexity of the speech signal. This study was the first to use a more appropriate baseline: spectrally rotated speech. Spectral rotation involves flipping the frequencies of speech around an axis such that high frequencies become low, and vice versa. This renders speech unintelligible but maintains spectral and temporal structure. The original Positron Emission Tomography (PET) study employed an elegant factorial design in which participants listened to clear and noise-vocoded speech (an intelligible speech stimulus with reduced spectral detail), and their unintelligible rotated equivalents. This isolated neural responses associated with speech comprehension by contrasting the response to clear and noise-vocoded speech with the average of the unintelligible rotated equivalents and spectral detail by comparing the average of clear and rotated speech to their noise-vocoded equivalents. Activity was found in the left anterior superior temporal sulcus (STS) for speech comprehension and in the right superior temporal gyrus (STG) for spectral detail. Further, regions of the left posterior superior temporal cortex showed elevated activity to intelligible clear and noise-vocoded speech, and unintelligible rotated speech, in the context of reduced activity to rotated noise-vocoded speech. Given that clear, noise-vocoded and rotated speech contain acoustic-phonetic information, while rotated noise-vocoded does not, this provided evidence for a hierarchical processing pathway that transformed acoustic–phonetic information to meaningful speech along a posterior-anterior axis. This fit well with work in non-human primates suggesting multiple streams of processing in the brain, including a hierarchically organized, anteriorly directed sound-to-meaning pathway (Rauschecker, 1998; Kaas and Hackett, 1999; Rauschecker and Tian, 2000; Tian et al., 2001).

A later functional Magnetic Resonance Imaging (fMRI) replication found elevated activity in left anterior STS to intelligible speech, as well as in the posterior part of the sulcus (Narain et al., 2003). The authors applied the ***global null*** conjunction (Price and Friston, 1997) which identified conjoint effects for the two simple intelligibility contrasts: [clear speech–rotated speech] and [noise-vocoded-rotated noise-vocoded speech], by testing for regions in which there was an averaged effect of intelligibility, in the absence of differences between these effects. This suggested a common mechanism for processing different forms of intelligible speech. However, the fixed effects analyses, used in this and the previous study, did not allow inferences to be extended to the wider population.

Another fMRI replication by Okada et al. (2010) conducted random effects analyses extending inferences beyond the tested participants. They found activity predominantly within lateral temporal cortex for the averaged response to intelligible speech, with bilateral activity found in the anterior and posterior superior temporal cortex. The authors also conducted multivariate pattern analyses (MVPA) (O'Toole et al., 2007; Mur et al., 2009; Pereira et al., 2009). This approach considers the pattern of activity over multiple voxels, allowing weakly discriminative information to be pooled over multiple data points, affording, in some instances, greater sensitivity (Haynes and Rees, 2006). Neural patterns were first normalized to remove the mean signal for each trial; ensuring that the MVPA analysis did not recapitulate the results of the univariate analysis. Using this approach, Okada et al. showed that intelligible speech could be discriminated from unintelligible sounds within regions of interest (ROIs) in early auditory cortex. This was unexpected within the context of hierarchical accounts of speech perception, in which early auditory regions engage in acoustic, rather than higher order language functions, and given that rotated speech was thought to be a close acoustic match to speech. A more expected finding was that bilateral anterior and posterior temporal ROIs successfully discriminated between intelligible and unintelligible speech. In an effort to identify regions that were sensitive to intelligibility in the absence of sensitivity to acoustics, Okada et al. expressed accuracies for intelligibility classifications relative to those for spectral detail, to create an “acoustic invariance” metric. This showed that the left posterior and right mid temporal cortex differed to primary auditory cortex on this metric, suggesting a more intelligibility selective response in these regions. Notably, however, the authors did not directly compare the strength of univariate responses between temporal lobe regions, nor did they examine multivariate responses beyond the superior temporal cortex.

The final replication by Evans et al. (2014) also combined univariate and multivariate analyses. The univariate main effect of intelligibility was associated with bilateral activity within lateral temporal cortex, spreading along the STS from posterior to anterior in the left and from mid to anterior in the right hemisphere. Only the left anterior STS was significantly activated by both simple effects, this time testing for the ***conjunction null*** (Nichols et al., 2005) rather than the more liberal ***global null conjunction***. Follow up tests indicated that the left anterior STS showed the strongest univariate intelligibility response. MVPA analyses were conducted using a searchlight technique (Kriegeskorte et al., 2006), in which classification was conducted iteratively on small patches across the entire brain. The authors elected not to use an acoustic invariance metric, as Okada and colleagues had done, because they noted that noise-vocoded speech differs from clear speech in both intelligibility and spectral detail, making the measure difficult to interpret. Using this approach, successful classifications of intelligible speech were found in a much wider fronto-temporo-parietal network. Interestingly, when classification accuracies were compared within the same ROIs in which univariate activity had been compared, posterior rather than anterior STS regions showed the highest classification accuracies. This highlighted the possibility that there may be multiple ways in which intelligibility could be encoded and that this may differ in anterior versus posterior regions. Evans et al. (2014) also conducted a fully factorial univariate analysis, interrogating for the first time the interaction between intelligibility and spectral detail. This revealed that the right planum temporale responded more to rotated speech than to all other sounds. This was unexpected, given the assumption that the baseline would activate early auditory regions equivalently to speech. This result, alongside Okada et al.'s finding of sensitivity to intelligibility in and around Heschl's gyrus, emphasized the difficulty of finding an appropriate non-speech baseline.

So what have we gained from these studies? These investigations are successful replications; elevated univariate activity in response to intelligible speech was found in the left anterior temporal STS across all studies. In addition, these replications extended the initial findings by delineating a much broader fronto-temporo-parietal sentence processing network (Davis and Johnsrude, 2003; Rodd et al., 2005; Obleser et al., 2007; Friederici et al., 2010; Davis et al., 2011; Abrams et al., 2012; Adank, 2012), consistent with the notion of multiple, rather than a single, comprehension stream (Peelle et al., 2010). Indeed, converging evidence suggests that both anterior ***and*** posterior STS play an important role in resolving speech intelligibility and that the relative balance of importance depends on how it is measured. This might suggest that speech intelligibility is encoded at different spatial scales across the temporal cortices.

As well as revealing a broader intelligibility network, these replications raise important questions about non-speech baselines. Rotated speech has proven a useful tool to separate “low level” acoustic from “higher level” linguistic processes (Boebinger et al., 2015; Lima et al., 2015; McGettigan et al., 2015; Evans et al., 2016; Meekings et al., 2016). However the replications discussed here, unexpectedly, showed that primary auditory cortex could distinguish between rotated and clear speech, and that some neural regions responded selectively to rotation as compared to clear speech. Why might this occur? It may reflect differences in the acoustic profile of rotated speech. For example, spectral rotation of fricatives results in broadband high frequency energy that is pushed into low frequency regions, a feature not characteristic of speech. Equally, it may reflect the fact that early auditory areas are capable of higher order linguistic processing (Formisano et al., 2008; Kilian-Hutten et al., 2011) either by virtue of local responses or via co-activation with higher order language regions. Taking a broader perspective, these findings demonstrate the difficulty of synthesizing non-speech baselines with the same acoustic properties as speech. Indeed, philosophically, the search for the perfect baseline is doomed to failure as the best baseline is speech itself. This, in combination with recent behavioral studies suggesting intermediate representations between speech-specific and more general acoustic processes (Iverson et al., 2016) call into question the logic of speech-non-speech baseline subtraction. This is not to suggest that we abandon this approach altogether, but rather, highlights the need to integrate evidence across multiple baselines and methodological approaches. One such alternative is to exploit similarities and differences between different kinds of speech to separate linguistic from acoustic processes (Joanisse et al., 2007; Raizada and Poldrack, 2007; Correia et al., 2014; Evans and Davis, 2015).

What insights can we gain concerning replication from these neuroimaging studies? First, they highlight the difficulty of defining “successful” replication. Evidence in favor of replication in behavioral studies may be reduced to the presence or absence of an effect. This distinction is much more complex in neuroimaging as multiple hypotheses are tested at tens of thousands of measurement points. Indeed, how similar do two statistical brain maps have to be to constitute a successful replication? Further, the complex data collection and analysis pipelines involved in functional neuroimaging likely reduce the likelihood of successful replication. Indeed, given this, it is surprising how similar the results are across the studies described. Second, these studies highlight that successful replications can provide new knowledge and highlight the role that methodological advancements can play in that process. Indeed, much less would have been gained from replicating the original study as it had been first performed. In this instance, advances in analysis played a crucial role in providing new insights on brain function, and upon the experimental paradigm itself. In this respect, given the fast pace of methodological change, neuroimaging arguably has the most to gain from replication going forward.

## Author contributions

The author confirms being the sole contributor of this work and approved it for publication.

### Conflict of interest statement

The author declares that the research was conducted in the absence of any commercial or financial relationships that could be construed as a potential conflict of interest.

## References

[B1] Abrams D. A. Ryali S. Chen T. Balaban E. Levitin D. J. Menon V. (2012). Multivariate activation and connectivity patterns discriminate speech intelligibility in Wernicke's, Broca's, and Geschwind's areas. Cereb. Cortex. 23, 1703–1714. 10.1093/cercor/bhs165 22693339 PMC3673181

[B2] Adank P. (2012). The neural bases of difficult speech comprehension and speech production: Two Activation Likelihood Estimation (ALE) meta-analyses. Brain Lang. 122, 42–54. 10.1016/j.bandl.2012.04.014 22633697

[B3] Arsenault J. S. Buchsbaum B. R. (2016). No evidence of somatotopic place of articulation feature mapping in motor cortex during passive speech perception. Psychon. Bull. Rev. 23, 1231–1240. 10.3758/s13423-015-0988-z 26715582

[B4] Barch D. M. Yarkoni T. (2013). Introduction to the special issue on reliability and replication in cognitive and affective neuroscience research. Cogn. Affect. Behav. Neurosci. 13, 687–689. 10.3758/s13415-013-0201-7 23922199

[B5] Bench J. Kowal A. Bamford J. (1979). The BKB (Bamford-Kowal-Bench) sentence lists for partially-hearing children. Br. J. Audiol. 13, 108–112. 10.3109/03005367909078884 486816

[B6] Bennett C. M. Miller M. B. (2013). fMRI reliability: influences of task and experimental design. Cogn. Affect. Behav. Neurosci. 13, 690–702. 10.3758/s13415-013-0195-1 23934630

[B7] Boebinger D. Evans S. Rosen S. Lima C. F. Manly T. Scott S. K. (2015). Musicians and non-musicians are equally adept at perceiving masked speech. J. Acoust. Soc. Am. 137, 378–387. 10.1121/1.4904537 25618067 PMC4434218

[B8] Button K. S. Ioannidis J. P. Mokrysz C. Nosek B. A. Flint J. Robinson E. S.. (2013). Power failure: why small sample size undermines the reliability of neuroscience. Nat. Rev. Neurosci. 14, 365–376. 10.1038/nrn3475 23571845

[B9] Carp J. (2012). The secret lives of experiments: methods reporting in the fMRI literature. Neuroimage 63, 289–300. 10.1016/j.neuroimage.2012.07.004 22796459

[B10] Carp J. (2013). Better living through transparency: improving the reproducibility of fMRI results through comprehensive methods reporting. Cogn. Affect. Behav. Neurosci. 13, 660–666. 10.3758/s13415-013-0188-0 23839069

[B11] Correia J. Formisano E. Valente G. Hausfeld L. Jansma B. Bonte M. (2014). Brain-based translation: fMRI decoding of spoken words in Bilinguals reveals language-independent semantic representations in anterior temporal lobe. J. Neurosci. 34, 332–338. 10.1523/JNEUROSCI.1302-13.2014 24381294 PMC6608172

[B12] Davis M. H. Ford M. A. Kherif F. Johnsrude I. S. (2011). Does semantic context benefit speech understanding through “Top Down” processes? Evidence from time-resolved sparse fMRI. J. Cogn. Neurosci. 23, 3914–3932. 10.1162/jocn_a_00084 21745006

[B13] Davis M. H. Johnsrude I. S. (2003). Hierarchical processing in spoken language comprehension. J. Neurosci. 23, 3423–3431. 12716950 10.1523/JNEUROSCI.23-08-03423.2003 PMC6742313

[B14] Eickhoff S. B. Laird A. R. Fox P. M. Lancaster J. L. Fox P. T. (2017). Implementation errors in the GingerALE Software: description and recommendations. Hum. Brain Mapp. 38, 7–11. 10.1002/hbm.23342 27511454 PMC5323082

[B15] Eklund A. Nichols T. E. Knutsson H. (2016). Cluster failure: why fMRI inferences for spatial extent have inflated false-positive rates. Proc. Natl. Acad. Sci. U.S.A. 113:201602413. 10.1073/pnas.1602413113 27357684 PMC4948312

[B16] Evans S. Davis M. H. (2015). Hierarchical organization of auditory and motor representations in speech perception: evidence from searchlight similarity analysis. Cereb. Cortex 25, 4772–4788. 10.1093/cercor/bhv136 26157026 PMC4635918

[B17] Evans S. Kyong J. S. Rosen S. Golestani N. Warren J. E. McGettigan C.. (2014). The pathways for intelligible speech: multivariate and univariate perspectives. Cereb. Cortex 24, 2350–2361. 10.1093/cercor/bht083 23585519 PMC4128702

[B18] Evans S. McGettigan C. Agnew Z. K. Rosen S. Scott S. K. (2016). Getting the cocktail party started: masking effects in speech perception. J. Cogn. Neurosci. 28, 483–500. 10.1162/jocn_a_00913 26696297 PMC4905511

[B19] Formisano E. De Martino F. Bonte M. Goebel R. (2008). “Who” Is Saying “What”? brain-based decoding of human voice and speech. Science 322, 970–973. 10.1126/science.1164318 18988858

[B20] Friederici A. D. Kotz S. A. Scott S. K. Obleser J. (2010). Disentangling syntax and intelligibility in auditory language comprehension. Hum. Brain Mapp. 31, 448–457. 10.1002/hbm.20878 19718654 PMC6870868

[B21] Gorgolewski K. J. Auer T. Calhoun V. D. Craddock R. C. Das S. Duff E. P.. (2016a). The brain imaging data structure, a format for organizing and describing outputs of neuroimaging experiments. Sci. Data 3, 160044. 10.1038/sdata.2016.44 27326542 PMC4978148

[B22] Gorgolewski K. J. Varoquaux G. Rivera G. Schwartz Y. Sochat V. V. Ghosh S. S.. (2016b). NeuroVault.org: a repository for sharing unthresholded statistical maps, parcellations, and atlases of the human brain. Neuroimage 124, 1242–1244. 10.1016/j.neuroimage.2015.04.016. 25869863 PMC4806527

[B23] Haynes J. D. Rees G. (2006). Decoding mental states from brain activity in humans. Nat. Rev. Neurosci. 7, 523–534. 10.1038/nrn1931 16791142

[B24] Iverson P. Wagner A. Rosen S. (2016). Effects of language experience on pre-categorical perception: distinguishing general from specialized processes in speech perception. J. Acoust. Soc. Am. 139, 1799. 10.1121/1.4944755 27106328

[B25] Joanisse M. F. Zevin J. D. McCandliss B. D. (2007). Brain mechanisms implicated in the preattentive categorization of speech sounds revealed using fMRI and a short-interval habituation trial paradigm. Cereb. Cortex 17, 2084–2093. 10.1093/cercor/bhl124 17138597

[B26] Kaas J. H. Hackett T. A. (1999). “What” and “where” processing in auditory cortex. Nat. Neurosci. 2, 1045–1047. 10.1038/15967 10570476

[B27] Kilian-Hutten N. Valente G. Vroomen J. Formisano E. (2011). Auditory cortex encodes the perceptual interpretation of ambiguous sound. J. Neurosci. 31, 1715–1720. 10.1523/JNEUROSCI.4572-10.2011 21289180 PMC6623724

[B28] Kriegeskorte N. Goebel R. Bandettini P. (2006). Information-based functional brain mapping. Proc. Natl. Acad. Sci. U.S.A. 103, 3863–3868. 10.1073/pnas.0600244103 16537458 PMC1383651

[B29] Lima C. F. Lavan N. Evans S. Agnew Z. Halpern A. R. Shanmugalingam P.. (2015). Feel the noise: relating individual differences in auditory imagery to the structure and function of sensorimotor systems. Cereb. Cortex 25, 4638–4650. 10.1093/cercor/bhv134 26092220 PMC4816805

[B30] McGettigan C. Walsh E. Jessop R. Agnew Z. K. Sauter D. A. Warren J. E.. (2015). Individual differences in laughter perception reveal roles for mentalizing and sensorimotor systems in the evaluation of emotional authenticity. Cereb. Cortex 25, 246–257. 10.1093/cercor/bht227 23968840 PMC4259281

[B31] Meekings S. Evans S. Lavan N. Boebinger D. Krieger-Redwood K. Cooke M.. (2016). Distinct neural systems recruited when speech production is modulated by different masking sounds. J. Acoust. Soc. Am. 140, 8–19. 10.1121/1.4948587 27475128

[B32] Mur M. Bandettini P. A. Kriegeskorte N. (2009). Revealing representational content with pattern-information fMRIan introductory guide. Soc. Cogn. Affect. Neurosci. 4, 101–109. 10.1093/scan/nsn044 19151374 PMC2656880

[B33] Narain C. Scott S. K. Wise R. J. S. Rosen S. Leff A. Iversen S. D.. (2003). Defining a left-lateralized response specific to intelligible speech using fMRI. Cereb. Cortex 13, 1362–1368. 10.1093/cercor/bhg083 14615301

[B34] Nichols T. Brett M. Andersson J. Wager T. Poline J. B. (2005). Valid conjunction inference with the minimum statistic. Neuroimage 25, 653–660. 10.1016/j.neuroimage.2004.12.005 15808966

[B35] Obleser J. Wise R. J. Dresner M. A. Scott S. K. (2007). Functional integration across brain regions improves speech perception under adverse listening conditions. J. Neurosci. 27, 2283–2289. 10.1523/JNEUROSCI.4663-06.2007 17329425 PMC6673469

[B36] Okada K. Rong F. Venezia J. Matchin W. Hsieh I. H. Saberi K.. (2010). Hierarchical organization of human auditory cortex: evidence from acoustic invariance in the response to intelligible speech. Cereb. Cortex 20, 2486–2495. 10.1093/cercor/bhp318 20100898 PMC2936804

[B37] Open Science Collaboration (2015). Estimating the reproducibility of psychological science. Science 349:aac4716. 10.1126/science.aac4716 26315443

[B38] O'Toole A. J. Jiang F. Abdi H. Pénard N. Dunlop J. P. Parent M. A. (2007). Theoretical, statistical, and practical perspectives on pattern-based classification amoroaches to the analysis of functional neuroimaging data. J. Cogn. Neurosci. 19, 1735–1752. 10.1162/jocn.2007.19.11.1735 17958478

[B39] Peelle J. E. Johnsrude I. S. Davis M. H. (2010). Hierarchical processing for speech in human auditory cortex and beyond. Front. Hum. Neurosci. 4:51. 10.3389/fnhum.2010.00051 20661456 PMC2907234

[B40] Pereira F. Mitchell T. Botvinick M. (2009). Machine learning classifiers and fMRI: a tutorial overview. Neuroimage 45, S199–S209. 10.1016/j.neuroimage.2008.11.007 19070668 PMC2892746

[B41] Pernet C. Poline J.-B. (2015). Improving functional magnetic resonance imaging reproducibility. Gigascience 4, 15. 10.1186/s13742-015-0055-8 25830019 PMC4379514

[B42] Poldrack R. A. Baker C. I. Durnez J. Gorgolewski K. J. Matthews P. M. Munafò M. R.. (2017). Scanning the horizon: towards transparent and reproducible neuroimaging research. Nat. Rev. Neurosci. 18, 115–126. 10.1038/nrn.2016.167 28053326 PMC6910649

[B43] Poldrack R. A. Barch D. M. Mitchell J. P. Wager T. D. Wagner A. D. Devlin J. T.. (2013). Toward open sharing of task-based fMRI data: the OpenfMRI project. Front. Neuroinform. 7:12. 10.3389/fninf.2013.00012 23847528 PMC3703526

[B44] Poldrack R. A. Fletcher P. C. Henson R. N. Worsley K. J. Brett M. Nichols T. E. (2008). Guidelines for reporting an fMRI study. Neuroimage 40, 409–414. 10.1016/j.neuroimage.2007.11.048 18191585 PMC2287206

[B45] Price C. J. Friston K. J. (1997). Cognitive conjunction: a new approach to brain activation experiments. Neuroimage 5, 261–270. 10.1006/nimg.1997.0269 9345555

[B46] Pulvermüller F. Huss M. Kherif F. Moscoso del Prado Martin F. Hauk O. Shtyrov Y. (2006). Motor cortex maps articulatory features of speech sounds. Proc. Natl. Acad. Sci. U.S.A. 103, 7865–7870. 10.1073/pnas.0509989103 16682637 PMC1472536

[B47] Raizada R. D. S. Poldrack R. A. (2007). Selective amplification of stimulus differences during categorical processing of speech. Neuron 56, 726–740. 10.1016/j.neuron.2007.11.001 18031688

[B48] Rauschecker J. P. (1998). Cortical processing of complex sounds. Curr. Opin. Neurobiol. 8, 516–521. 10.1016/S0959-4388(98)80040-8 9751652

[B49] Rauschecker J. P. Scott S. K. (2009). Maps and streams in the auditory cortex: nonhuman primates illuminate human speech processing. Nat. Neurosci. 12, 718–724. 10.1038/nn.2331 19471271 PMC2846110

[B50] Rauschecker J. P. Tian B. (2000). Mechanisms and streams for processing of “what” and “where” in auditory cortex. Proc. Natl. Acad. Sci. U.S.A. 97, 11800–11806. 10.1073/pnas.97.22.11800 11050212 PMC34352

[B51] Rodd J. M. Davis M. H. Johnsrude I. S. (2005). The neural mechanisms of speech comprehension: fMRI studies of semantic ambiguity. Cereb. Cortex 15, 1261–1269. 10.1093/cercor/bhi009 15635062

[B52] Scholar.google.com. (2017). Sophie Scott - Google Scholar Citations. Available online at: https://scholar.google.com/citations?user=qyUbUn0AAAAJ (Accessed January 23, 2017).

[B53] Schmidt S. (2009). Shall we really do it again? The powerful concept of replication is neglected in the social sciences. Rev. Gen. Psychol. 13, 90–100. 10.1037/a0015108

[B54] Schomers M. R. Pulvermüller F. (2016). Is the sensorimotor cortex relevant for speech perception and understanding? an integrative review. Front. Hum. Neurosci. 10:435. 10.3389/fnhum.2016.00435 27708566 PMC5030253

[B55] Scott S. K. Blank C. C. Rosen S. Wise R. J. S. (2000). Identification of a pathway for intelligible speech in the left temporal lobe. Brain 123, 2400–2406. 10.1093/brain/123.12.2400 11099443 PMC5630088

[B56] Scott S. K. Johnsrude I. S. (2003). The neuroanatomical and functional organization of speech perception. Trends Neurosci. 26, 100–107. 10.1016/S0166-2236(02)00037-1 12536133

[B57] Scott S. K. Wise R. J. S. (2004). The functional neuroanatomy of prelexical processing in speech perception. Cognition 92, 13–45. 10.1016/j.cognition.2002.12.002 15037125

[B58] Shou H. Eloyan A. Lee S. Zipunnikov V. Crainiceanu A. N. Nebel M. B.. (2013). Quantifying the reliability of image replication studies: the image intraclass correlation coefficient (I2C2). Cogn. Affect. Behav. Neurosci. 13, 714–724. 10.3758/s13415-013-0196-0 24022791 PMC3869880

[B59] Simmons J. P. Nelson L. D. Simonsohn U. (2011). False-positive psychology: undisclosed flexibility in data collection and analysis allows presenting anything as significant. Psychol. Sci. 22, 1359–1366. 10.1177/0956797611417632 22006061

[B60] Szucs D. Ioannidis J. P. A. (2016). Empirical assessment of published effect sizes and power in the recent cognitive neuroscience and psychology literature. bioRxiv. 10.1101/071530 PMC5333800 28253258

[B61] Tian B. Reser D. Durham A. Kustov A. Rauschecker J. P. (2001). Functional specialization in rhesus monkey auditory cortex. Science 292, 290–293. 10.1126/science.1058911 11303104

[B62] Turner B. O. Miller M. B. (2013). Number of events and reliability in fMRI. Cogn. Affect. Behav. Neurosci. 13, 615–626. 10.3758/s13415-013-0178-2 23754543

